# Cortisol and β-Endorphin Responses During a Two-Month Exercise Training Program in Patients with an Opioid Use Disorder and on a Substitution Treatment

**DOI:** 10.3390/ijms26115178

**Published:** 2025-05-28

**Authors:** Alexandros E. Psarianos, Anastassios Philippou, Argyro Papadopetraki, Eirini Chatzinikita, Costas Chryssanthopoulos, Apostolos Theos, Athanasios Theocharis, Chara Tzavara, Thomas Paparrigopoulos

**Affiliations:** 1 1st Department of Psychiatry, Medical School, National and Kapodistrian University of Athens, 11528 Athens, Greece; htzavara@med.uoa.gr (C.T.); tpaparrig@med.uoa.gr (T.P.); 2 Greek Organization Against Drugs (OKANA), 10433 Athens, Greece; ath.theocharis@gmail.com; 3 Department of Physiology, Medical School, National and Kapodistrian University of Athens, 11527 Athens, Greece; tfilipou@med.uoa.gr (A.P.); argpapa@med.uoa.gr (A.P.); echatzin@med.uoa.gr (E.C.); chryssan@phed.uoa.gr (C.C.); 4 Section of Sports Medicine, Department of Community Medicine & Rehabilitation, Umeå University, 901 87 Umeå, Sweden; apostolos.theos@umu.se

**Keywords:** cortisol, β-endorphin, physical exercise, opioid substitution treatment, opioid use disorder

## Abstract

Physical exercise may affect drug use by balancing neurohormonal system mechanisms. Cortisol and β-endorphin, associated with stress, mood, and pleasure feelings, can be affected by exercise and act as regulators of withdrawal symptoms associated with drug use during short-term abstinence. The present study investigated the effect of a supervised, two-month moderate-intensity aerobic exercise program on salivary cortisol and β-endorphin levels in patients with an opioid use disorder (OUD) and on a substitution treatment during a short-term, 24–36 h withdrawal phase from methadone/buprenorphine medication. Ninety opioid users (41 females) in methadone and buprenorphine substitution treatment were randomly divided into four groups: (a) buprenorphine exercise (BEX) (n = 26; age (mean ± SD): 41.9 ± 6.1 yrs), (b) buprenorphine control (BCON) (n = 25; age: 41.9 ± 5.6 yrs), (c) methadone exercise (MEX) (n = 20; age: 46.7 ± 6.6 yrs), and (d) methadone control (MCON) (n = 19; age: 46.1 ± 7.5 yrs). The exercise intervention groups (BEX and MEX) followed a training program on a treadmill for 20 min at 70% HRmax, 3 days/week for 8 weeks. The responses of cortisol and β-endorphin were measured before (t0) and immediately after an exercise session (t20) on different days (i.e., the 1st, 12th, and 24th session) corresponding to the beginning, middle, and end of the training program. A significant increase in β-endorphin levels was observed after the completion of the training intervention (24th exercise session) in both exercise groups (BEX before: 63.8 ± 33; BEX after: 185.6 ± 182.8 pg/mL; MEX before: 115 ± 211; MEX after: 262.3 ± 505.7 pg/mL), whereas β-endorphin was decreased in the control groups (BCON before: 34.7 ± 20.1; BCON after: 24.2 ± 8.8 pg/mL; MCON before: 129.7 ± 185.7; MCON after: 84.9 ± 104.3 pg/mL) (*p* < 0.05). Inversely, cortisol decreased in both exercise groups post-intervention (BEX before: 9.5 ± 5.9; BEX after: 2.8 ± 1.5 ng/mL; MEX before: 9.3 ± 6.6; MEX after: 3.1 ± 1.5 ng/mL) and increased in control groups (BCON before: 6.3 ± 2.5; BCON after: 10.1 ± 5.4 ng/mL; MCON before: 7.5 ± 3.2; MCON after: 12.5 ± 4.3 ng/mL) (*p* < 0.05). Moderate-intensity aerobic exercise can beneficially influence β-endorphin and cortisol levels in individuals undergoing treatment for OUD. By increasing endogenous opioid levels and reducing stress hormones, exercise emerges as a promising adjunctive strategy for alleviating withdrawal symptoms, enhancing emotional regulation, and potentially reducing the risk of relapse. The inverse relationship between β-endorphin and cortisol highlights the role of physical activity as a long-term modulator of neuroendocrine function in the context of substance use recovery. Future research should prioritize longitudinal studies extending beyond two months and involving larger, more diverse populations. Additionally, investigating the integration of exercise with non-pharmacological interventions—and its effects on relapse rates, mental health outcomes, and overall quality of life—would provide further insight into its therapeutic value in addiction recovery.

## 1. Introduction

Long-term adherence to opioid substitution therapy is often limited due to the persistent neurobiological and physiological disruptions caused by chronic opioid use [1,2]. These disruptions affect systems involved in pleasure, anxiety, and overall hedonic balance [3,4]. However, aerobic exercise has demonstrated potential in reversing some of these changes, influencing hormones like cortisol and β-endorphin, which are key regulators of stress and mood [3,5,6,7]. Physical activity thus emerges as a promising complementary approach for supporting recovery in individuals with substance use disorders [8].

The neurohormonal system, particularly the hypothalamic–pituitary–adrenal (HPA) axis, plays a central role in addiction and relapse. Opioid use disrupts this axis, especially during withdrawal, leading to abnormal hormone responses, notably elevated cortisol levels [3,4]. This dysregulation contributes to cycles of anxiety and dysphoria, which reinforce drug-seeking behavior and relapse. During opioid use, the HPA axis is suppressed, resulting in low cortisol levels [3,4]. However, in early withdrawal, cortisol spikes due to hyperactivation of the HPA axis, although levels tend to normalize with prolonged abstinence [9,10].

Exercise is a controlled form of stress that activates the HPA axis, leading to cortisol release. Over time, regular physical activity results in decreased cortisol responses during both exercise and psychosocial stress [11,12,13]. This adaptation builds stress resilience, which may be particularly beneficial for individuals recovering from opioid dependence [14,15]. The effect of exercise on cortisol is intensity dependent. Low- to moderate-intensity aerobic exercise tends to reduce cortisol levels, while higher-intensity workouts increase them [16]. Because withdrawal is marked by elevated cortisol [17,18,19,20], moderate physical activity may help mitigate symptoms like insomnia, pain, and psychological distress by reducing cortisol secretion [21,22,23].

The endogenous opioid β-endorphin is another key neurohormone affected by opioid use and withdrawal, with analgesic and mood-enhancing properties [24]. It interacts mainly with μ- and δ-opioid receptors and plays a role in reward, pain regulation, and immune response [25]. Short-acting opioids and antagonists like naloxone lower β-endorphin levels [26], and while substitution therapies help restore them, they often remain suboptimal [26]. Low β-endorphin levels during early abstinence are linked to heightened craving and relapse risk [24,27].

Exercise significantly influences β-endorphin levels [28,29], especially at moderate-to-high intensities [30] and durations exceeding five minutes [31,32,33]. This rise in β-endorphin potentially contributes to improved mood and reduced craving, acting as a protective mechanism against relapse. Further, the reinforcing effects of β-endorphin during exercise may promote continued participation in physical activity, enhancing overall treatment outcomes [34].

The present study investigated the effect of a supervised, two-month moderate-intensity aerobic exercise program on salivary cortisol and β-endorphin levels in patients with an opioid use disorder (OUD) and on a substitution treatment during a short-term 24–36 h withdrawal phase from methadone/buprenorphine medication.

## 2. Results

### 2.1. Sociodemographic and Clinical Characteristics of the Participants

The sample included 90 participants. Fifty-one (56.7%) were receiving buprenorphine, while the remaining 39 (43.3%) were on methadone. Among the 51 participants taking buprenorphine, 26 (51%) were in the exercise group, and 25 (49%) were in the control group. Of the 39 participants on methadone, 20 (51.3%) were in the exercise group, and 19 (48.7%) were in the control group. Participants’ demographic characteristics, along with information about their substance use, are presented in Table 1. No significant differences were found between the exercise and control groups, regardless of the substitution treatment. When comparing the two substitution treatments within the exercise groups, it was noted that participants using methadone were significantly older, had a higher body mass index (BMI), began substance use at a younger age, and experienced more relapses during rehabilitation. Additionally, those who did not attend the follow-up showed a higher rate of recurrence in the use of either opioids or non-opioid substances.

### 2.2. Medical History

Table 1 shows the medical histories of the participants. Most of the participants exhibited both psychiatric and substance use disorders, with this comorbidity being consistent across all groups and both substitution treatments. Specifically, according to the medical histories recorded at OKANA and based on DSM-5 criteria, participants presented with the following conditions: (a) mental and behavioral disorders due to multiple drug use and other psychoactive substances (F.19-N = 24); (b) mood disorders (F.30 and F.39-N = 6); (c) schizophrenia, schizotypal, and delusional disorders (F.20 and F.29-N = 3); (d) neurotic, stress-related, and somatoform disorders (F.40 and F.48-N = 22); (e) panic disorders (episodic paroxysmal anxiety, F.41, N = 24); and (f) mild depressive episodes (F.32-N = 3). Overall, high rates of anxiety and depression were the most common disorders among these individuals. Of the 90 volunteers in this study, 51 (56.7%) were taking buprenorphine, and 39 (43.3%) were on methadone. Half of this population reported experiencing depression at some point in their lives, and nearly one-third reported having a depressed mood during treatment (see Table 1).

### 2.3. Measurements of β-Endorphin in the Buprenorphine and Methadone Subgroups

**Figure 1:**
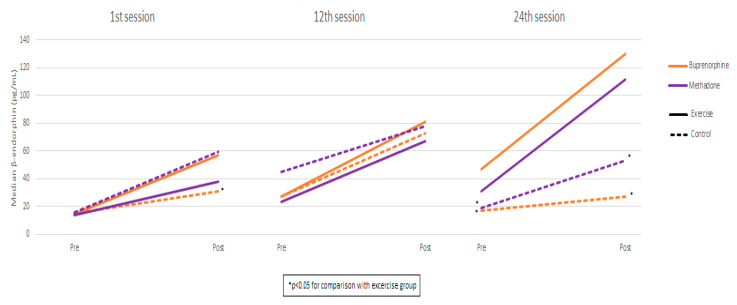
β-endorphin measurements by session, group, and substitution treatment.

Measurements and comparisons (with the level of statistical significance) between study groups of salivary β-endorphin in the buprenorphine and methadone subgroups at the three timepoints are presented in Table 2 and Figure 1.

### 2.4. β-Endorphin: Buprenorphine Exercise Group vs. Methadone Exercise Group

Comparing the buprenorphine exercise group with the methadone exercise group, β-endorphin values were found to be similar in both pre-and post-exercise measurements in all sessions. Also, the degree-of-endorphin increase was similar between exercise groups in all sessions. Using mixed linear regression models, it was found that, in the exercise groups, β-endorphin significantly increased from pre- to post-measurement, and the degree of increase was similar across the three sessions (as the interaction terms were not found to be significant) (Table 3). Also, it was found that, during the 24th session, β-endorphin values were significantly lower compared to the 1st and 12th sessions. These results apply both to the buprenorphine and the methadone group.

### 2.5. Measurements of Cortisol in the Buprenorphine and Methadone Subgroups

**Figure 2:**
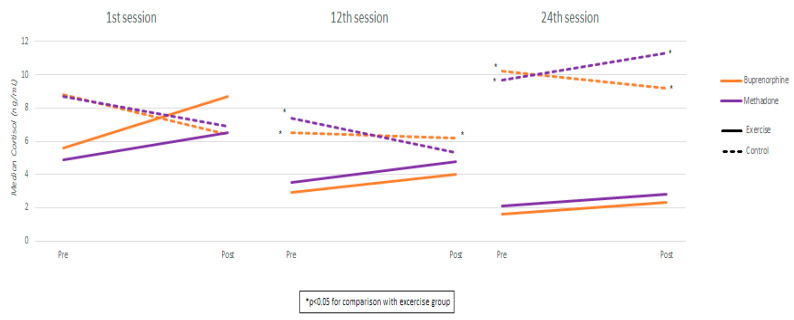
Cortisol measurements by session, group, and substitution treatment.

Measurements and comparisons (with the level of statistical significance) between study groups of salivary cortisol in the buprenorphine and methadone subgroups at the three timepoints are presented in Table 4 and Figure 2.

### 2.6. Cortisol: Buprenorphine Exercise Group vs. Methadone Exercise Group

Comparing the buprenorphine exercise group with the methadone exercise group, cortisol values were found to be similar in both pre- and post-exercise measurements in all sessions. Also, the degree of cortisol increase was similar between exercise groups in all sessions. Using mixed linear regression models, it was found that, in the exercise groups, cortisol significantly increased from pre- to post-measurement, and the degree of increase was similar across the three sessions (as the interaction terms were not found to be significant) (Table 5). Also, it was found that, during the 24th session, cortisol values were significantly higher compared to the 1st and 12th sessions. These results apply both to the buprenorphine and the methadone group.

### 2.7. Correlation Between β-Endorphin and Cortisol

**Figure 3:**
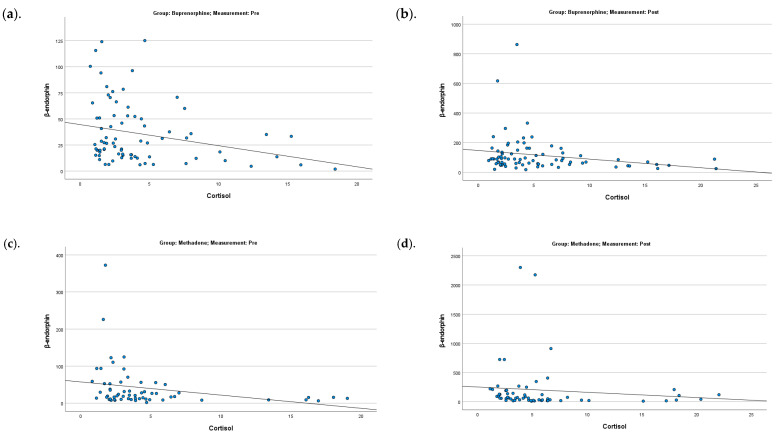
Correlation between β-endorphin and cortisol for the buprenorphine (**a**,**b**) pre- and post-measurements) and methadone (**c**,**d**) pre- and post-measurements) groups.

Significant negative correlations between β-endorphin and cortisol values were found in both pre- (for the buprenorphine group: rho = −0.25 and *p* = 0.026; for the methadone group: rho = −0.42 and *p* = 0.001) and post-measurements (for the buprenorphine group: rho = −0.28 and *p* = 0.015; for the methadone group: rho = −0.36 and *p* = 0.005), regardless of control/exercise group and session (Figure 3).

## 3. Discussion

### 3.1. Changes in β-Endorphin and Cortisol Levels

This study revealed significant changes in β-endorphin and cortisol levels among individuals with an OUD and on a substitution treatment participating in a structured exercise program. Notably, β-endorphin levels were significantly higher in the exercise groups compared to controls, in both buprenorphine and methadone treatment cohorts. These differences were evident after the 1st session and both before and after the 24th session. The buprenorphine exercise group exhibited a steady increase in β-endorphin levels, with significantly higher values in the 24th session compared to the 1st and 12th sessions. In contrast, the control group showed a temporary increase by the 12th session, followed by a return to baseline. The methadone exercise group followed a consistent upward trend, reaching significance after one month, while the control group showed no meaningful changes.

These results align with prior research indicating that individuals with OUD exhibit lower baseline β-endorphin levels compared to healthy individuals, likely due to the 24 h abstinence period prior to testing [18,35]. Such low levels may contribute to increased craving, possibly due to a deficit in the endogenous opioid system. This hypothesis, supported by Koob (2021), suggests that individuals with OUD seek exogenous opioids to mitigate the negative emotional state known as “hyperkatifeia” [18,35,36]. Earlier findings by Marchesi et al. (1997) and Roschina et al. (2021) indicate that reduced β-endorphin levels in early withdrawal stages play a role in relapse by enhancing the desire to use opioids as a form of negative reinforcement [27,37].

Cortisol levels, measured across sessions, also revealed significant differences. In both treatment groups, participants in the exercise group exhibited markedly lower cortisol levels than controls during the 12th and 24th sessions. In the buprenorphine group, cortisol steadily declined from the 1st to the 24th session. The control group showed an initial drop by the 12th session, followed by an increase by the 24th session, returning to baseline levels. A similar significant trend was observed in the methadone group, whereas the control group’s cortisol levels increased.

These elevated baseline cortisol levels are consistent with the previous literature, which links increased cortisol to early withdrawal symptoms, anxiety, and cognitive impairment [38,39]. Studies by Errico et al. (2002) and others have reported prolonged hyperactivity of the HPA axis in individuals abstaining from opioids or other substances, with elevated salivary cortisol persisting for up to 25 days post-cessation [40,41,42,43]. This heightened cortisol response to psychosocial stress appears to be a core characteristic of the chronic stress profile in OUD, often manifesting as elevated morning cortisol levels that fail to normalize through typical feedback mechanisms [44].

### 3.2. Stress, β-Endorphin, and Cortisol: A Dynamic Interaction

The combination of low β-endorphin and elevated cortisol levels observed before the exercise program highlights a significant neurohormonal imbalance in individuals with OUD during early abstinence [24,37,38,45]. This imbalance, consistent with the existent literature, may play a critical role in relapse vulnerability, as both hormones are intimately linked to stress regulation and reward mechanisms [45,46]. As our findings demonstrate, exercise has the potential to restore this balance by reducing cortisol levels and increasing β-endorphin, thereby mitigating withdrawal symptoms and reducing craving intensity.

Throughout the exercise intervention, a noteworthy inverse relationship emerged between β-endorphin and cortisol. Specifically, while β-endorphin levels steadily increased across sessions, cortisol levels declined. This pattern was statistically significant in both buprenorphine (*p* = 0.026) and methadone (*p* = 0.001) groups before exercise and post-exercise (*p* = 0.015 and *p* = 0.005, respectively). These findings suggest that, with consistent exercise over time, the neuroendocrine system adapts in a way that favors stress regulation, potentially through the long-term upregulation of β-endorphin, which appears to suppress cortisol activity.

This regulatory role is further supported by the physiological interplay between the anterior pituitary, where β-endorphin is synthesized, and the broader HPA axis. Exercise, especially when repeated and sustained, acts as a controlled stressor that activates and gradually stabilizes this system. The resulting hormonal adaptations not only enhance mood and reduce stress reactivity but may also reduce vulnerability to substance use by reshaping brain circuits involved in reward, motivation, and emotional regulation.

### 3.3. Therapeutic Implications of Exercise in OUD

Our study contributes to a growing body of evidence supporting physical activity as a therapeutic tool in managing OUD. Aerobic exercise—defined as sustained, rhythmic movement of large muscle groups requiring increased cardiovascular and respiratory effort—emerged as the most effective modality. It was associated with both immediate and sustained improvements in neurohormonal balance and psychological well-being. Moderate-intensity aerobic exercise (approximately 70% of maximum heart rate) has been shown to reduce cortisol levels, increase β-endorphin, and promote neuroplastic changes in key brain areas such as the nucleus accumbens and prefrontal cortex.

Reviews by Bardo et al. (2015) and Psarianos et al. (2023) support this mechanism, noting that regular exercise may activate the hippocampal–prefrontal–amygdala circuitry, leading to increased reward sensitivity, enhanced emotional control, and reduced stress responsiveness [3,47]. This neurobiological remodeling provides a plausible explanation for the observed improvements in our study and supports the integration of aerobic exercise into comprehensive treatment plans for individuals with substance use disorders.

In contrast, participants in the control groups did not exhibit similar improvements. Initial changes in hormone levels may have provided temporary relief, but these effects were not sustained. Thus, by the end of the second month, β-endorphin levels had decreased, and cortisol levels had risen to or above baseline, contributing to increased anxiety and discomfort. This emphasizes the necessity of regular, ongoing exercise to achieve lasting neurobiological benefits.

The therapeutic significance of this study lies in its objectives to the following: (a) standardize physical exercise protocols, (b) integrate these protocols into OKANA’s therapeutic service network (via the therapeutic contract), and (c) prescribe exercise to patients as part of their treatment plan, tailored to their specific stage in the therapeutic process. Through rigorous methodological implementation and evidence-based validation, this approach may enrich the overall treatment framework of OUD patients and act as a supplementary treatment strategy in the rehabilitation process.

## 4. Materials and Methods

### 4.1. Study Design, Participants, Recruitment, and Experimental Procedures

This study is part of a randomized controlled trial conducted in adult patients with OUDs. The recruitment, inclusion, stratified randomization of the subjects, and the experimental procedures are described in detail in a previously published paper [48]. The study was conducted under the supervision of specialized healthcare providers for opioid-dependent patients. The study design ensured the following: (a) an accessible location with adequate space for exercise and a safe natural environment, including equipment for managing potential overdoses or, in rare cases, aggressive behavior on-site and (b) access to patients’ medical records, in collaboration with the OKANA’s therapeutic services. This access facilitated the recruitment and supervision of volunteers participating in the exercise program, the evaluation of diagnoses, therapeutic histories, and social and psychiatric status, as well as the maintenance of fully updated patient records. Supervision of the exercise program was critical to its effectiveness, as it resulted in greater improvements in participants’ overall physical condition compared to unsupervised programs. Support and commitment from staff and exercise instructors have been shown to enhance motivation, increase participation, and reduce dropout rates. Furthermore, supervised exercise training is particularly advantageous for individuals with dependency issues, offering both greater efficacy and enhanced safety relative to unsupervised alternatives. Regarding the participants in the control groups, they did not engage in exercise; they received standard care and were provided only with educational information about the health benefits of exercise, delivered in a relevant setting. Additionally, they were informed that they would have the opportunity to participate in exercise sessions at a later stage of the study. Participants from each group were unable to interact with those in other groups, as they met at different times and locations.

### 4.2. Ethical Approval

The study was conducted in accordance with the Declaration of Helsinki and was approved by the Ethics Committee of the Medical School of the National and Kapodistrian University of Athens (approval number: 141-27/06/2020). Approval was also obtained from the Greek Organization Against Drugs (OKANA), as it was conducted within its service premises (approval number: 21550-31/05/2019). All participants signed a written informed consent.

### 4.3. Analysis of Salivary Cortisol and β-Endorphin

Salivary cortisol and β-endorphin were determined before and after exercise intervention in all study groups. Saliva samples were obtained prior to exercise (PRE) and immediately after exercise (IP). Sampling was performed on three different exercise days (1st, 12th, and 24th day of intervention) representing the beginning, middle, and end of the exercise program. The measurements were conducted following 24–36 h of abstinence from the last administration of the substitute medication. Subjects had to refrain from consuming any food, hot beverages, or brushing their teeth for two hours prior to arrival. Upon arrival, participants were asked to remain seated for 15 min before providing the resting sample (PRE-sample). Approximately 2 mL of saliva were collected using special vials (Salivettes SARSTEDT AG & Co. KG, 51588, Numbrecht, GERMANY) and stored at −80 °C until assay. Concentrations of β-endorphin and cortisol were assessed via commercially available, enzyme-linked immunosorbent assays ELISA, (Cortisol ELISA Kit, KA1885, Abnova, Taipei, Taiwan, and Human Β-endorphin ELISA Kit-Colorimetric, NBP2-78774, Novus Biologicals, Centennial CO, USA) and a spectrophotometer (SpectraMax M3, Molecular Devices, Sunnyvale, CA, USA). To eliminate inter-assay variance, all samples were thawed once and analyzed in duplicate in the same assay run by a single investigator, with an average coefficient of variation (CV) of 9.92% for β-endorphin and 9.76% for cortisol.

### 4.4. Aerobic Exercise Training

The exercise training program was implemented within the therapeutic service premises of OKANA, a treatment center, where beneficiaries receive their substitution treatment. The aerobic exercise protocol was conducted on a treadmill (20 min duration at 70% HRmax at 3 times per week for 8 weeks), tailored to each participant’s fitness level. HRmax was estimated using the following formula: 220 − age × 0.7. The treadmill speed was determined by progressively increasing the speed and monitoring the HR. Sessions began with a resting HR measurement, a 5 min warm-up, and 3 min familiarization at 80% of the target speed, followed by 20 min of aerobic exercise. The heart rate and perceived exertion (Borg RPE scale) were recorded regularly. Each session concluded with a 5 min cooldown involving breathing and stretching exercises. More details are provided in a previously publish paper [48].

### 4.5. Statistical Analysis

Quantitative variables were tested for normality using the Kolmogorov–Smirnov criterion, and they were expressed as mean values (standard deviation) and as the median (interquartile range), while categorical variables were expressed as absolute and relative frequencies. The Mann–Whitney test was used for the comparison of β-endorphin and cortisol between two groups. Effect sizes from Mann–Whitney test were computed based on the formula r = z/√N, where z is the Mann–Whitney test statistic value and N is the sample size. This effect size uses the Cohen’s interpretation guidelines of 0.1–0.29 (small effect), 0.3–0.49 (moderate effect), and ≥0.5 (large effect) [49]. The Friedman test was used for the comparison of β-endorphin and cortisol across all sessions. The Bonferroni correction was used in case of multiple testing to control for type I error. Spearman correlations coefficients (rho) were used to explore the association between β-endorphin and cortisol. Mixed linear models were used for investigating the changes in β-endorphin and cortisol between the measurements and among the sessions. Adjusted regression coefficients (β) with standard errors (SE) were computed from the results of the mixed models. The logarithmic transformations of the dependent variables were used in the mixed linear models. All reported *p*-values are two-tailed. Statistical significance was set at *p* < 0.05, and analyses were conducted using SPSS statistical software (version 26.0).

## 5. Limitations of the Study

While our findings are promising, several limitations must be acknowledged. First, the study’s relatively short duration limits insights into the long-term sustainability of the hormonal adaptations observed. Second, the sample size was modest, and there were occasional challenges with adherence to the saliva collection protocol. Variability in participants’ baseline fitness and lifestyle factors also introduced potential confounders. Additionally, individuals who opted into the exercise program may have been more intrinsically motivated, possibly influencing the outcomes. Also, all participants were receiving pharmacological treatment for OUD, which may limit generalizability to untreated populations or those undergoing alternative therapies.

Finally, a key methodological limitation of the present study is the inherent difficulty in implementing a double-blind design in exercise-based interventions. Unlike pharmacological trials, where the use of placebo controls is feasible, blinding participants in exercise studies is virtually impossible, as individuals are aware of whether they are engaging in physical activity. This awareness may introduce expectancy effects; for example, participants in the exercise group may experience psychological benefits—such as improved mood, a sense of accomplishment, or increased motivation—simply due to their perception of engaging in a beneficial activity. In contrast, control group participants may feel disappointed or excluded, potentially increasing stress and negatively influencing outcomes. These differing subjective experiences may partially confound observed hormonal and psychological outcomes, including changes in cortisol and β-endorphin levels. Several research teams have sought to address this challenge through alternative approaches, such as implementing ‘sham exercise’ protocols (e.g., stretching or very low-intensity movements perceived as exercise), incorporating attention-matched control activities, or using deception regarding the purpose of the study arms. However, these strategies raise ethical and logistical concerns, especially when involving vulnerable populations such as individuals with OUD. For example, Abrantes et al. (2019) discuss the limitations and partial solutions to minimizing bias in behavioral interventions involving exercise [50].

Future studies could benefit from the inclusion of additional psychological assessments—such as expectancy questionnaires or measures of perceived benefit—to help disentangle the physiological effects of exercise from its psychological components. Moreover, the introduction of a third comparator group receiving non-exercise interventions (e.g., mindfulness training) could provide a more robust control condition, allowing for a clearer evaluation of the unique contributions of physical activity.

## 6. Conclusions

This study demonstrates that moderate-intensity aerobic exercise can beneficially modulate β-endorphin and cortisol levels in individuals undergoing treatment for OUD. By increasing endogenous opioids and reducing stress hormones, exercise offers a powerful adjunctive strategy for managing withdrawal symptoms, improving emotional regulation, and decreasing relapse risk. The inverse relationship between β-endorphin and cortisol underscores the potential of physical activity as a long-term regulator of neuroendocrine function in substance use recovery. Future research should focus on longitudinal studies that extend beyond two months and include larger, more diverse participant groups. Exploring the integration of exercise with non-pharmacological interventions and examining its impact on relapse rates, mental health outcomes, and quality of life would further clarify its role in addiction recovery.

## Figures and Tables

**Table 1:** Sociodemographic and clinical characteristics of the participants.

		Buprenorphine		*p* ^1^	Methadone		*p* ^1^	*p* ^2^
		Exercise Group	Control Group		Exercise Group	Control Group		
		Ν = 26; 28.9%	Ν = 25; 27.8%		Ν = 20; 22.2%	Ν = 19; 21.1%		
		Ν (%)	Ν (%)		Ν (%)	Ν (%)		
Age (years), mean (SD)		41.9 (6.1)	41.9 (5.6)	0.983 +	46.7 (6.6)	46.1 (7.5)	0.794 +	0.014 +
Gender								
	Men	15 (57.7)	11 (44.0)	0.328 ++	12 (60.0)	11 (57.9)	0.894 ++	0.875 ++
	Women	11 (42.3)	14 (56.0)		8 (40.0)	8 (42.1)		
BMI (kg/m^2^), mean (SD)		23 (2.4)	22.9 (2.3)	0.911 +	25 (3.4)	25.3 (1.8)	0.742 +	0.019 +
BMI (kg/m^2^)								
	Normal	22 (84.6)	16 (64.0)	0.091 ++	9 (45.0)	8 (42.1)	0.855 ++	0.004 ++
	Overweight/Obese	4 (15.4)	9 (36.0)		11 (55.0)	11 (57.9)		
Age at first use, mean (SD)		21 (2.9)	22.2 (3.3)	0.175 +	17.5 (2.5)	18.2 (3.2)	0.449 +	<0.001 +
Diagnosis								
	F.19	5 (19.2)	2 (8.0)	-	10 (50.0)	7 (36.8)	-	-
	F.19–F.30	0 (0.0)	0 (0.0)		1 (5.0)	0 (0.0)		
	F.19–F.32	0 (0.0)	0 (0.0)		1 (5.0)	2 (10.5)		
	F.19–F.40	1 (3.8)	0 (0.0)		3 (15.0)	2 (10.5)		
	F.19–F.41.1	2 (7.7)	2 (8.0)		0 (0.0)	0 (0.0)		
	F.20–7.29	0 (0.0)	0 (0.0)		1 (5.0)	2 (10.5)		
	F.30–F.39	0 (0.0)	0 (0.0)		1 (5.0)	2 (10.5)		
	F.40–F.48	5 (19.2)	10 (40.0)		3 (15.0)	4 (21.1)		
	F.41	5 (19.2)	2 (8.0)		0 (0.0)	0 (0.0)		
	F.41	7 (26.9)	7 (28.0)		0 (0.0)	0 (0.0)		
	F.41–F.39	1 (3.8)	2 (8.0)		0 (0.0)	0 (0.0)		
HIV		3 (11.5)	0 (0.0)	0.080 ++	6 (30.0)	2 (10.5)	0.235 ‡‡	0.149 ‡‡
Hepatitis		3 (11.5)	4 (16.0)	0.703 ‡‡	4 (20.0)	0 (0.0)	0.106 ‡‡	0.682 ‡‡
Number of relapses during rehabilitation, mean (SD), median (IQR)		1 (1–2)	1 (1–2)	0.802 ‡	4 (3–4)	4 (3–4)	0.767 ‡	<0.001 ‡

+ Student’s *t*-test. ‡ Mann–Whitney test. ++ Pearson’s chi-square test. ‡‡ Fisher’s exact test. *p* ^1^—value for comparison between exercise and control group. *p* ^2^—value for comparison between buprenorphine exercise group and methadone exercise group.

**Table 2:** β-endorphin measurements by group and substitution treatment at the three timepoints.

β-Endorphin (pg/mL)	1st Session			12th Session			24th Session		
	Pre	Post	Change	Pre	Post	Change	Pre	Post	Change
Buprenorphine									
Control									
Mean (95% CI)	16 (14.5–17.5)	34.7 (26.3–43)	18.6 (10.7–26.6)	38.8 (27.4–50.2)	82.1 (55.1–109.2)	43.4 (19.5–67.2)	18.3 (15.7–20.8)	24.2 (20.5–27.8)	5.9 (3.5–8.2)
Median (IQR)	14.8 (13.4–16.7)	30.6 (16.1–57.1)	14 (3.1–40.4)	26.8 (21.9–45.5)	73.1 (35–98.2)	27.6 (2.1–64.6)	17.1 (13.6–19.2)	26.8 (17.4–29.3)	6 (1–10.7)
Exercise									
Mean (95% CI)	18.4 (13.1–23.7)	63.8 (50.5–77.1)	45.4 (34.1–56.7)	34.1 (25–43.2)	95.3 (73.2–117.3)	61.2 (43.4–79)	55.5 (40.9–70)	185.6 (111.8–259.5)	130.2 (57.6–202.7)
Median (IQR)	14.5 (7.2–31.3)	57.2 (42.3–86.2)	40.6 (26–63)	26.9 (14.8–53)	81.1 (55.9–124.5)	49.4 (28.1–91.4)	46.7 (25.4–78.5)	129.8 (89–204.4)	67.9 (40.6–139.7)
*p* +	0.665	0.001	<0.001	0.522	0.250	0.020	<0.001	<0.001	<0.001
r ^1^	−0.06	−0.49	−0.52	−0.09	−0.16	−0.32	−0.68	−0.86	−0.85
Methadone									
Control									
Mean (95% CI)	17.4 (14.8–20)	129.7 (40.2–219.2)	112.3 (24.6–200)	54.4 (36.4–72.4)	187.9 (76.8–299.1)	133.5 (39.2–227.8)	20.1 (17.3–22.9)	84.9 (34.6–135.2)	64.8 (15.4–114.2)
Median (IQR)	15.7 (12.7–19.4)	59.6 (24.2–160.5)	43.9 (7.5–132.4)	44.7 (24.1–81.6)	77.8 (55.2–247.9)	41.6 (24.5–166.3)	18.5 (14.5–24.7)	53.1 (19.1–108.1)	32.5 (2.2–76.3)
Exercise									
Mean (95% CI)	19.4 (9.7–29.2)	115 (16.2–213.7)	95.6 (0.6–190.5)	34.9 (19.6–50.1)	220.8 (−7–448.5)	185.9 (−36–407.9)	67.2 (25.6–108.8)	262.3 (25.7–499)	195.1 (−27–417.3)
Median (IQR)	13.8 (7.9–19.6)	37.9 (17.8–96.9)	22.7 (9.8–76.2)	23.5 (10.9–51.6)	66.9 (26.9–165.4)	48.2 (13.5–123.3)	31.1 (17.6–81.9)	111.6 (61.8–216.3)	62.4 (32.2–132.7)
*p* +	0.185	0.448	0.749	0.056	0.399	0.555	0.033	0.013	0.025
r ^1^	−0.21	−0.12	−0.05	−0.31	−0.14	−0.09	−0.34	−0.40	−0.36
*p* ++	0.877	0.215	0.268	0.520	0.506	0.756	0.352	0.506	0.723
r ^1^	−0.02	−0.18	−0.16	−0.09	−0.10	−0.05	−0.14	−0.10	−0.05

+ *p*-value for comparison between exercise and control group (Mann–Whitney test). ++ *p*-value for comparison between buprenorphine exercise group and methadone exercise group (Mann–Whitney test). ^1^ Effect size from Mann–Whitney test based on formula r = z/√N.

**Table 3:** Mixed linear regression results for exercise groups with β-endorphin as the dependent variable.

Dependent Variable: β-Endorphin		β +	SE ++	*p*
Buprenorphine subgroup	Measurement (Post vs. Pre)	1.38	0.12	<0.001
	Session			
	1st vs. 24th	0.65	0.12	<0.001
	12th vs. 24th	1.16	0.13	<0.001
	Measurement ∗ Session interaction			
	1st vs. 24th	−0.27	0.17	0.109
	12th vs. 24th	−0.25	0.17	0.139
Methadone subgroup	Measurement (Post vs. Pre)	1.25	0.15	<0.001
	Session			
	1st vs. 24th	0.59	0.15	<0.001
	12th vs. 24th	1.05	0.15	<0.001
	Measurement ∗ Session interaction			
	1st vs. 24th	−0.09	0.21	0.687
	12th vs. 24th	−0.12	0.21	0.586

Note. Analysis was completed after having logarithmically transformed the dependent variable, and it included only the exercise groups. + regression coefficient. ++ Standard error of regression coefficient.

**Table 4:** Cortisol measurements by group and substitution treatment at the three timepoints.

Cortisol (ng/mL)	1st Session			12th Session			24th Session		
	Pre	Post	Change	Pre	Post	Change	Pre	Post	Change
Buprenorphine									
Control									
Mean (95% CI)	7.9 (6.8–9)	6.3 (5.3–7.3)	−1.6 (−2–−1.3)	6.3 (5.5–7.1)	5.4 (4.6–6.2)	−0.9 (−1.2–−0.6)	11.4 (8.7–14)	10.1 (7.8–12.3)	−1.3 (−2–−0.6)
Median (IQR)	8.8 (6.3–9.5)	6.4 (4.4–8.4)	−1.7 (−2.1–−1.4)	6.5 (5.2–7.6)	6.2 (4.7–6.5)	−0.9 (−1.4–−0.5)	10.2 (7.2–15)	9.2 (5.7–13.1)	−1.4 (−2.1–−0.3)
Exercise									
Mean (95% CI)	7.4 (5.4–9.4)	9.5 (7.2–11.9)	2.1 (0.7–3.5)	3.3 (2.5–4)	4.2 (3.3–5.1)	0.9 (0.5–1.3)	2 (1.6–2.4)	2.8 (2.2–3.4)	0.8 (0.5–1.1)
Median (IQR)	5.6 (3.6–10.5)	8.7 (4.3–13.6)	1.3 (0.3–1.9)	2.9 (1.8–4.1)	4 (2.2–5.8)	0.6 (0.2–1.1)	1.6 (1.2–2.5)	2.3 (1.7–3.5)	0.6 (0.2–1.3)
*p* +	0.258	0.083	<0.001	<0.001	0.040	<0.001	<0.001	<0.001	<0.001
r ^1^	−0.16	−0.24	−0.86	−0.66	−0.29	−0.86	−0.84	−0.77	−0.73
Methadone									
Control									
Mean (95% CI)	8.7 (7.3–10.2)	7.5 (6–9.1)	−1.2 (−2–−0.4)	7.5 (6.2–8.8)	5.6 (4.4–6.9)	−1.8 (−2.9–−0.8)	11.3 (9.3–13.3)	12.5 (10.4–14.6)	1.2 (0–2.4)
Median (IQR)	8.7 (6.3–10.2)	6.9 (4.4–9.4)	−0.8 (−1–−0.3)	7.4 (5.2–9.3)	5.3 (3.2–7.2)	−1.9 (−2.8–−0.2)	9.7 (8.3–13.9)	11.3 (8.9–14)	1.7 (0.1–2.5)
Exercise									
Mean (95% CI)	8 (5.2–10.8)	9.3 (6.2–12.4)	1.3 (0.9–1.7)	3.7 (2.9–4.5)	5.3 (3.6–7)	1.6 (0.4–2.8)	2.4 (1.9–2.9)	3.1 (2.4–3.8)	0.7 (0.4–1)
Median (IQR)	4.9 (3.5–14.7)	6.5 (4.4–16.2)	1.2 (0.6–1.8)	3.5 (2.2–5.1)	4.8 (2.7–6.1)	1.1 (0.5–1.5)	2.1 (1.7–3.1)	2.8 (1.9–4)	0.5 (0.3–0.7)
*p* +	0.086	0.888	<0.001	<0.001	0.431	<0.001	<0.001	<0.001	0.149
r ^1^	−0.27	−0.02	−0.86	−0.66	−0.13	−0.74	−0.86	−0.86	−0.23
*p* ++	0.790	0.842	0.833	0.278	0.268	0.163	0.141	0.358	0.947
r ^1^	−0.04	−0.03	−0.03	−0.16	−0.16	−0.21	−0.22	−0.14	−0.01

+ *p*-value for comparison between exercise and control group (Mann–Whitney test). ++ *p*-value for comparison between buprenorphine exercise group and methadone exercise group (Mann–Whitney test). ^1^ Effect size from Mann–Whitney test based on formula r = z/√N.

**Table 5:** Mixed linear regression results for exercise groups with cortisol as the dependent variable.

Dependent Variable: Cortisol		β +	SE ++	*p*
Buprenorphine subgroup	Measurement (Post vs. Pre)	0.25	0.07	<0.001
	Session			
	1st vs. 24th	−0.74	0.09	<0.001
	12th vs. 24th	−1.21	0.13	<0.001
	Measurement ∗ Session interaction			
	1st vs. 24th	0.001	0.10	0.991
	12th vs. 24th	0.06	0.01	0.556
Methadone subgroup	Measurement (Post vs. Pre)	0.19	0.07	0.012
	Session			
	1st vs. 24th	−0.59	0.10	<0.001
	12th vs. 24th	−1.03	0.14	<0.001
	Measurement ∗ Session interaction			
	1st vs. 24th	0.12	0.10	0.268
	12th vs. 24th	0.06	0.10	0.546

Note. Analysis was completed after having logarithmically transformed the dependent variable, and it included only the exercise groups. + regression coefficient ++ Standard error of regression coefficient.

## Data Availability

All data included in this study are available upon request by contact with the corresponding author.
